# Efficacy of Ciprofloxacin-Gentamicin Combination Therapy in Murine Bubonic Plague

**DOI:** 10.1371/journal.pone.0052503

**Published:** 2012-12-20

**Authors:** Nadine Lemaître, Isabelle Ricard, Elizabeth Pradel, Benoît Foligné, René Courcol, Michel Simonet, Florent Sebbane

**Affiliations:** 1 Laboratoire de Bactériologie-Hygiène, Centre Hospitalier Universitaire, Lille, France; 2 Université Lille Nord de France, Lille, France; 3 Université Lille 2, Lille, France; 4 INSERM U1019, Lille, France; 5 Centre National de la Recherche Scientifique UMR8204, Lille, France; 6 Institut Pasteur, Lille, France; University of Helsinki, Finland

## Abstract

Potential benefits of combination antibiotic therapy for the treatment of plague have never been evaluated. We compared the efficacy of a ciprofloxacin (CIN) and gentamicin (GEN) combination therapy with that of each antibiotic administered alone (i) against *Yersinia pestis in vitro* and (ii) in a mouse model of bubonic plague in which animals were intravenously injected with antibiotics for five days, starting at two different times after infection (44 h and 56 h). *In vitro*, the CIN+GEN combination was synergistic at 0.5x the individual drugs’ MICs and indifferent at 1x- or 2x MIC. *In vivo*, the survival rate for mice treated with CIN+GEN was similar to that observed with CIN alone and slightly higher than that observed for GEN alone 100, 100 and 85%, respectively when treatment was started 44 h post challenge. 100% of survivors were recorded in the CIN+GEN group *vs* 86 and 83% in the CIN and GEN groups, respectively when treatment was delayed to 56 h post-challenge. However, these differences were not statistically significant. Five days after the end of treatment, *Y. pestis* were observed in lymph nodes draining the inoculation site (but not in the spleen) in surviving mice in each of the three groups. The median lymph node log_10_ CFU recovered from persistently infected lymph nodes was significantly higher with GEN than with CIN (5.8 *vs.* 3.2, *p = *0.04) or CIN+GEN (5.8 *vs.* 2.8, *p = *0.01). Taken as the whole, our data show that CIN+GEN combination is as effective as CIN alone but, regimens containing CIN are more effective to eradicate *Y. pestis* from the draining lymph node than the recommended GEN monotherapy. Moreover, draining lymph nodes may serve as a reservoir for the continued release of *Y. pestis* into the blood – even after five days of intravenous antibiotic treatment.

## Introduction

The Gram-negative bacterium *Yersinia pestis* is responsible for plague, a zoonotic disease that primarily affects rodents and, to a lesser extent, humans [1]. *Yersinia pestis* is mostly transmitted to susceptible hosts by infected fleas. After the flea bite, *Y. pestis* migrates from the dermis and through the lymphatic vessels to regional lymph nodes, where rapid bacterial multiplication induces a tender, swollen feature (the bubo). Indeed, the bubo characterizes bubonic plague – the most common form of the disease. In some patients, the lymph node defenses are overwhelmed and the bacteria disseminate through the circulatory system, which causes life-threatening septicemia and (in some cases) secondary pneumonia. Patients developing secondary pneumonic plague can transmit the pathogen as an expectorated aerosol, with the newly contaminated patient developing primary pneumonic plague [1].

In the absence of early treatment, bubonic and pneumonic plagues are fatal in around 50% and 100% of cases, respectively [1]. On the basis of the *in vitro* antibiotic susceptibility of *Y. pestis*, animal studies and clinical experience with patients treated with antimicrobials during outbreaks of plague, aminoglycosides (such as streptomycin and gentamicin (GEN)) are the currently recommended first-choice antibiotics for human plague treatment. Cyclines (such as tetracycline and doxycycline) and ciprofloxacin are alternative treatment choices [2]–[7].

Animal studies of the efficacy of antibiotic therapy in plague are scarce. Most such studied have been performed in a mouse model of pneumonic plague because of threat of bioterrorism with contaminated aerosols [4], [6], [7]. In the one investigation to have used a mouse model of bubonic plague, the animals were subcutaneously inoculated with a *Y. pestis* load well over that transmitted by a flea bite (∼10^4^ CFU and 80 CFU, respectively) [8]. Under these experimental conditions (which lead to fulminant disease), antimicrobial chemotherapy was found only to be efficacious when initiated within 24 h of inoculation. Furthermore, the sterilizing activity of antibiotics was not assessed in the lymph node draining the inoculation site while this tissue is the first colonized by *Y. pestis* in bubonic model of plague [9], [10]. Lastly, the benefits of combination therapy for the treatment of plague have never been evaluated neither in preclinical experiments nor in clinical trials although combined antibiotic regimens are usually recommended for the treatment of serious Gram-negative infections [11].

We therefore studied the efficacy of treatment with GEN and ciprofloxacin (CIN) alone or in combination and initiated at different times (44 or 56 h) after intradermal inoculation of mice with a *Y. pestis* load similar to that injected by an infected flea.

## Materials and Methods

### Ethics Statement

Animals were housed in a dedicated facility at the Institut Pasteur de Lille (Lille, France), which is accredited by the French Ministry of Agriculture to perform experiments on live rodents (accreditation B59-350009) in compliance with the French and European regulations on the care and protection of laboratory animals (EC Directive 86/609 and the French Act #2001-486, issued on June 6, 2001). All Experiments were performed under the authorization number 59-350218. The French regulation does not require ethics approval by a specific committee. But, all animal experiments were authorized by French veterinary authorities and they were performed in compliance with the terms of the NIH Animal Welfare Insurance (#A5476-01, issued on 02/07/2007). All inoculations were performed under conditions to minimize any potential suffering and all effort was made to minimize animal suffering after bacterial inoculation. Animals were monitored twice a day during the course of treatment and then once a day for five days after injection of the last dose. Animals showing terminal signs of plague which is characterized by hunched posture, reluctance to move and to respond to external stimuli were sacrificed by overdose of isoflurane. At the end of the study, animals were euthanized with an overdose of isoflurane.

### Strain and Antibiotics

The virulent *Y. pestis* strain CO92 was used in the present study. For *in vitro* antibiotic susceptibility testing, CIN and GEN were purchased from Sigma-Aldrich (France). Stock solutions were obtained by dissolving powder in distilled water and were stored at –80°C until required. Dilutions in Mueller-Hinton (MH) broth were prepared from stock solutions. Sterile injectable formulations of CIN (a 2 mg/ml injection premix in a sterile, single-use container; Panpharma, France) and GEN sulfate (in a 40 mg/ml ampule; Panpharma, France) were used to treat infected animals.

### Antimicrobial Susceptibility Testing and the Kill-curve Assay

Determinations of Minimal Inhibitory Concentrations (MICs), Minimal Bactericidal Concentrations (MBCs) and checkerboard assays were performed according to the broth microdilution method in 96-well microtiter plates [12]. Briefly, an inoculum of 5×10^5^ CFU/ml was prepared from an overnight bacterial culture at 28°C in MH broth and was added to two-fold serial dilutions of CIN and GEN, alone or in combination. The final antibiotic concentrations ranged from 0.125 mg/L to 8 mg/L for GEN and 0.008 mg/L to 0.25 mg/L for CIN. Plates were incubated at 28°C and any detect bacterial growth 48 h later was noted with a mirror reader. The MIC was defined as the lowest drug concentration that completely inhibited *Y. pestis* growth at 48 h. The MBC was determined by taking 10 µl from wells without optically detected bacterial growth and streaking the volume onto a blood agar plate. Colonies were counted after 48 h of incubation at 28°C. The MBC was defined as the lowest concentration of antibiotic that killed 99.9% of the original inoculum.

For evaluation of the antimicrobial combination effect with a checkerboard assay, the fractional inhibitory concentrations of GEN (FIC_GEN_ = MIC_GEN_ when combined/MIC_GEN_ alone) and CIN (FIC_CIN_ = MIC_CIN_ when combined/MIC_CIN_ alone) were calculated. The fractional inhibitory concentration index (FICI) was calculated as FIC_GEN_+FIC_CIN_. The drug combination was considered to be synergistic for a FICI ≤0.5, indifferent for a FICI between 0.5 and 4 and antagonistic for a FICI >4 [13].

For the kill-curve assay, 5×10^5^ CFU/ml of *Y. pestis* from a culture growing exponentially in MH broth were added to 0.5x, 1x, and 2x MIC of CIN, GEN and CIN+GEN and incubated at 28°C with shaking. Aliquots were removed at regular time intervals and serially diluted in sterile phosphate-buffered saline (PBS) for counting viable cells. The limit of detection (LOD) for bacteria was ≤1.6 log_10_ CFU/ml. In preliminary experiments, the absence of antibiotic carry-over was checked by plating samples of bacterial suspension in the presence or absence of antibiotic dilutions [14]. Synergy and indifference were respectively defined as a ≥100- and <100-fold decrease in the colony count when the combination was compared with the most active single drug after 24 h of incubation. Antagonism was defined as a ≥100-fold increase in colony count when the combination was compared with the most active drug alone. All *in vitro* assays were performed in duplicate.

### Experimental Infection

Groups of 8- to 10-week-old-female OF1 mice (Charles River Laboratories, France) were intradermally (i.d.) inoculated in the upper right thigh with 100 CFU of *Y. pestis* previously grown at 21°C. This bacterial load approximates the average number of *Y. pestis* inoculated by flea, whereas 21°C corresponds to optimal temperature for blockage of the flea’s foregut proventriculus – an important event for optimal plague transmission by this vector to mammals [8]. At 44 h or 56 h post-challenge, daily intravenous (i.v.) injection of 300 µl of normal saline (placebo), CIN (30 mg/kg q12h), GEN (25 mg/kg q24h), or CIN+GEN (30 mg/kg q12h and 25 mg/kg q24h, respectively) into the tail vein was initiated. The treatment duration (5 days) and antibiotic doses were those usually used in previous experiments in mice [4], [6], [7]. Mouse mortality was recorded every 12 h during the course of treatment and then every 24 h for five days after injection of the last dose.

### Determination of the Bacterial Load in Tissues

Five days after the end of the treatment, the spleen and the inguinal lymph node draining the inoculation site from surviving mice were aseptically removed immediately after euthanasia, triturated through sterile mesh into 3 ml of sterile cold PBS and homogenized. Homogenates were plated on blood agar plates and CFU were counted after incubation at 28°C for 48 h. Bacteria titers were expressed as the log_10_ CFU per spleen or per lymph node. Sterile tissues contained ≤1.8 log_10_ CFU (i.e. below the LOD).

### Assay of the Serum Antibiotic Concentration

Venous blood was withdrawn by cardiac puncture from groups of 5 non-infected mice, 5 min. after i.v. antibiotic administration (i.e. the peak level) and prior to subsequent injection (i.e. the trough level). Serum levels of GEN were assayed in a fluorescent antibody assay, using the TdxFLX instrument (Abbott Laboratories) [15]. Serum levels of CIN were determined by high pressure liquid chromatography, using a NovaPack column (4 µm, 150×3.9 mm) with a mobile phase consisting of 9% acetonitrile in 0.1 M of H_3_PO_4_ (adjusted to pH 2 with tetrabutylammonium bromide), as previously described [16]. The eluent was monitored by UV absorption at 280 nm. Data was processed using KromaSystem 2000 software (version 1.83). The LOD was 0.1 and 0.25 mg/L for CIN and GEN, respectively.

### Statistical Analysis

The survival rates and sterilization tissue cultures in the different groups of mice were compared in a two-tailed Fischer’s exact test. Median bacterial counts in non-sterile tissues were compared in a Kruskall-Wallis test. The threshold for statistical significance was set to *p*≤0.05 for all tests.

## Results

### Antimicrobial Susceptibility Testing

**Figure 1:**
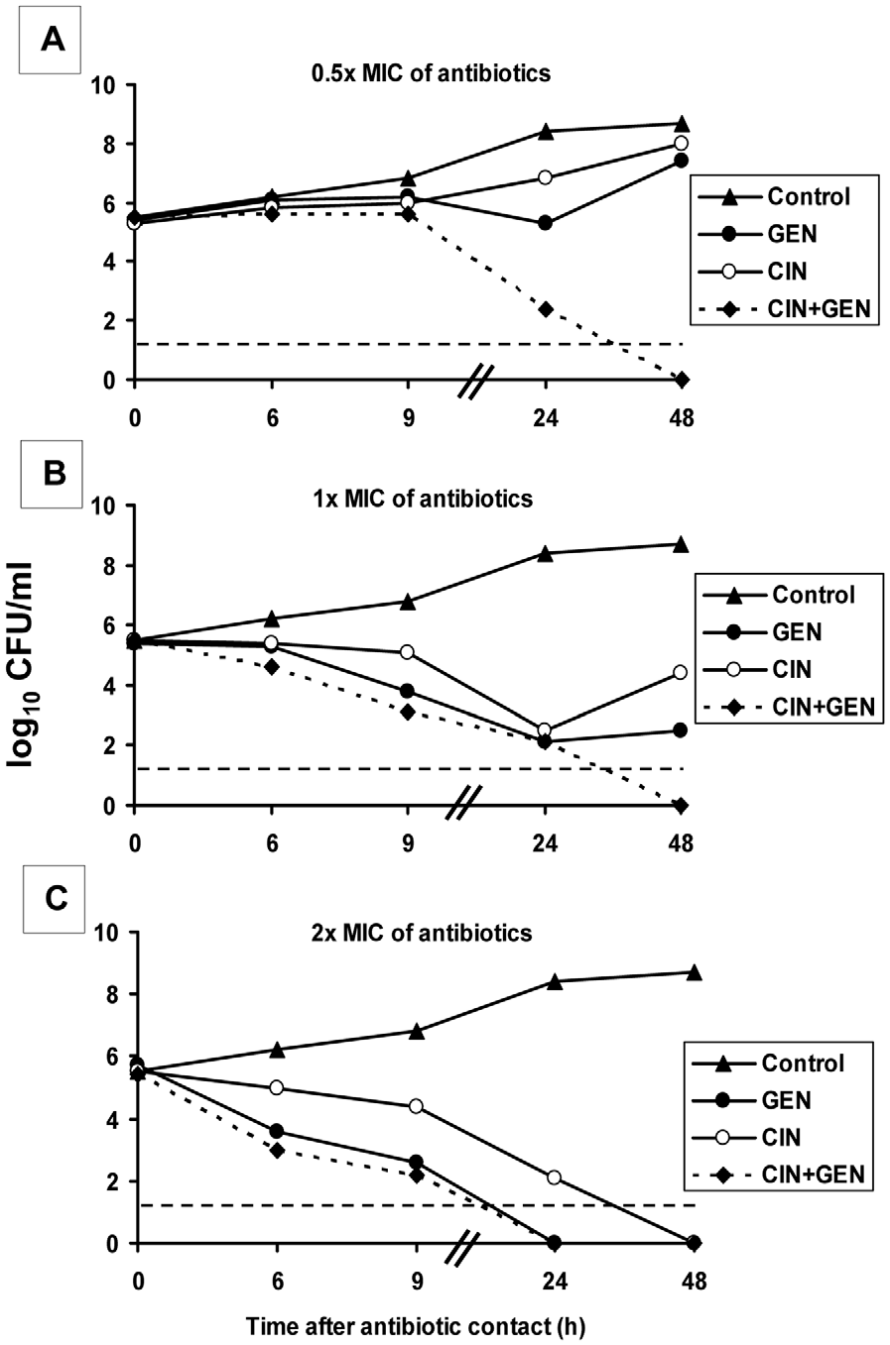
Activities of CIN and GEN in combination against *Y. pestis* CO92. CIN and GEN were respectively used at (A) 0.016 mg/L and 0.5 mg/L (0.5x MIC), (B) 0.032 mg/L and 1 mg/L (1x MIC) and (C) 0.064 mg/L and 2 mg/L (2x MIC). The dashed horizontal line indicates the LOD (≤1.6 log_10_ CFU).

The combination of CIN with aminoglycoside antibiotics on Gram-negative bacteria has been reported as indifferent, additive or synergistic, depending on the species tested [17]–[20]. We first assessed the antibacterial activity of CIN+GEN against *Y. pestis* CO92 *in vitro*. We confirmed that the MIC and MBC of CIN (0.032 and 0.064 mg/L, respectively) and GEN (1.0 and 2.0 mg/L, respectively) for the *Y. pestis* CO92 strain were similar to previously reported values [6], [21]. Next, by using the checkerboard microdilution method to assess synergy, we found that a combination of CIN and GEN was indifferent (FICI = 0.75). Lastly, we investigated the dynamic aspects of the antibiotics’ bactericidal activity (alone and when combined) in a kill-curve assay (Figure 1). At 0.5x MIC, the combination was synergistic after 24 h and prevented bacterial regrowth after 48 h of incubation (Figure 1A). At 1x MIC, both CIN and GEN alone were bactericidal after 24 h of incubation but did not prevent bacterial regrowth. At this concentration, the combination of CIN and GEN exhibited indifference but nevertheless prevented bacterial regrowth at 48 h (Figure 1B). Lastly, at 2x MIC (*i.e.* the MBC of both CIN and GEN), GEN was more rapidly bactericidal than CIN (after 10 h *vs.* 24 h of antibiotic exposure, respectively) and the antibiotic combination yielded much the same effects as the single drugs did (Figure 1C).

### Serum Antibiotic Concentrations

After a single i.v. injection, the mean peak plasma concentrations of CIN and GEN were 20±4 mg/L and 101±23.50 mg/L, respectively. Trough concentrations of CIN and GEN were not detectable. Thevalues of peak were greater than and trough concentration were equal to those achievable with the therapeutic doses used in humans [22], [23].

### Efficacy of Treatments Initiated 44 h after *Y. pestis* Inoculation

In an initial experiment, antibiotic therapy was initiated 44 h after the i.d. infectious challenge (Table 1, group A). None of the untreated mice (controls) survived after the challenge. Although not significant, the survival rates in the CIN+GEN and CIN groups, which were identical (19/19, 100%), were higher than the rate in the GEN group (17/20, 85%). No deaths were observed in either CIN+GEN or CIN groups of mice during the course of the experiment. In contrast, one and two mice treated with GEN alone succumbed during and after the 5-day antibiotic treatment period, respectively (Table 1).

**Table 1:** Survival of *Y. pestis*-infected mice treated with GEN alone, CIN alone and a combination of CIN+GEN.

	N° of mice at the following time point (days)^a^			
Experimental groups (N°)	0	1	5	10
**A**				
Control (20)	19	15	0	0
GEN (20)	20	20	19	17
CIN (19)	19	19	19	19
CIN+GEN (19)	19	19	19	19
**B**				
Control (20)	10	5	0	0
GEN (20)	12	12	10	10
CIN (20)	15	14	13	13
CIN+GEN (20)	14	14	14	14

Groups of 19–20 mice were i.d. infected with 100 CFU of *Y. pestis* and were given antibiotics i.v., either alone or in combination and once or twice daily (depending on the antimicrobial). The first dose of drugs was given 44 h (experiment A) or 56 h (experiment B) after the *Y. pestis* inoculation.

a Time point: 0, day of initiation of treatment; 1, one day after initiation of treatment; 5, the end of treatment-course; 10, euthanasia of survivors.

**Figure 2:**
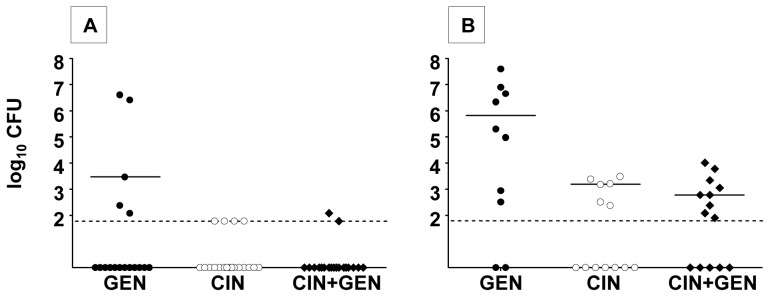
The effect of antibiotic treatment on bacterial load in the draining lymph node. Treatment with GEN at 25 mg/kg q24h (filled circles), CIN at 30 mg/kg q12h (open circles) and CIN at 30 mg/kg q12h in combination with GEN at 25 mg/kj q24h (filled diamonds) was initiated in mice 44 h (A) and 56 h (B) after the i.d challenge (100 CFU of *Y. pestis*). Individual bacterial counts in the draining lymph node 5 days after the end of the course regimen are shown. Median values are indicated by bars. The dashed horizontal line represents the LOD (≤1.8 log_10_CFU).

Determination of the bacterial load in surviving mice (regardless of the antimicrobial regimen used) revealed that all spleens (but not all lymph nodes draining the inoculation site) were sterile. In surviving mice treated with CIN+GEN, CIN alone and GEN alone, the rates of sterilization of lymph nodes were respectively 95% (18/19), 100% (19/19), and 70% (12/17), as presented in Figure 2. The bacterial load recovered from the sole infected lymph node in the CIN+GEN group was close to the LOD (2.1 log_10_ CFU). In the five persistently infected lymph nodes in the GEN group, the median log_10_ CFU count was 3.5 (range: 2.1–6.6). Thus, the sterilizing activity of the CIN+GEN combination was similar to that of CIN alone and slightly higher (*p = *0.08) than for GEN. Similarly, CIN alone was significantly more effective than GEN alone in eradicating *Y. pestis* from lymph nodes (*p = *0.02).

### Efficacy of Treatments Initiated 56 h after *Y. pestis* Inoculation

In the experiment described above, only one mouse died in the control group at time of treatment initiation (on day 0) – indicating that most of the mice were not yet seriously ill 44 h after bacterial inoculation. Therefore, in a second experiment, we looked at whether the CIN+GEN combination might be of value at a later stage in the infective process. To this end, antimicrobial chemotherapy was initiated 56 h post-challenge (Table 1, group B). In this context, the mortality rate reached 50% (10/20) in the control group at day 0; this finding confirmed that mice were in a more advanced disease stage than in the first experiment. Two mice in the GEN and CIN groups died during the treatment period– even though the animals had been receiving a complete regimen for several days. This contrasted with the absence of deaths over the same period in the combination therapy group (Table 1 group B). Finally, after the initiation of antimicrobial treatment, the survival rates in the mice treated with the combined regimen was higher (14/14, 100%) than in the CIN (13/15, 86%) and the GEN (10/12, 83%) groups, but it was not significant. Despite the longer time to initiation of antimicrobial chemotherapy, none of the spleens from surviving mice (regardless of the group) contained any detectable bacteria. In contrast, as shown in Figure 2B, the rate of lymph node sterilization for treatment initiation at 56 h was significantly lower (*p<*0.05) in each of the three treated groups than for initiation at 44 h (20% *vs.* 70%, 54% *vs.* 100% and 36% *vs.* 95% in the GEN, CIN and combination groups, respectively). The median number of CFU in non-sterilized lymph nodes in the combination treatment group (2.8; range: 1.9–4.0) was significantly lower (*p = *0.01) than that observed in the GEN group (median: 5.8; range: 2.5–7.6). Similarly, CIN produced a greater reduction of the bacterial load in the lymph node (median: 3.2; range: 2.4–3.5) than GEN alone (*p = *0.04).

Our findings indicated that the CIN+GEN combination was not more effective than CIN alone in terms of sterilizing spleens and draining lymph nodes at a later disease stage. However, we did find that regimens containing CIN (either alone or in combination with GEN) reduced the bacterial load in the draining lymph nodes more efficiently than a GEN-alone regimen did.

## Discussion

Herein, we sought to determine whether a CIN+GEN combination was more effective than either agent alone when administered to treat experimentally induced bubonic plague in the mouse. Indeed, the current standard treatment of plague is still based on the use of aminoglycoside monotherapy and although a combined antibiotic regimen with streptomycin, ciprofloxacin and ceftriaxone has been recently used to treat cases of human plague in China, its efficacy has not been compared with that of drugs administered singly [3], [5], [24]. Furthermore, in our model of bubonic plague, we challenged animals by intradermal route with a *Y. pestis* load similar to that transmitted by a flea-bite so that these experimental conditions are more physiologic than in the one previous preclinical study [7]. Indeed, the higher bacterial dose used led to a rapid and abnormal progression of the disease that need a prompt antimicrobial chemotherapy within 24 h of inoculation [7]. However, in human bubonic plague, bubo appears after a 2–8 days incubation period and thereby the antimicrobial chemotherapy is often not initiated within 24 h [1]. Therefore, in the present study we initiated the treatment 44 and 56 h after the challenge. because at these time-points, in rodents, clinical signs such as roughcast fur and limping due to lymphadenitis appear (10). It was shown that at 44 h post-inoculation, the draining lymph node is heavily colonized but bacterial load in blood is yet moderate and at 56 hours, septicemic phase is firmly established [9], [10]. Concerning the survival rate, our data revealed that CIN+GEN combination treatment was not superior to CIN or GEN monotherapies. In contrast, the eradication activity were higher with CIN-containing regimens than with GEN monotherapy, even though GEN is one of the first-choice drugs recommended for plague treatment [2], [3]. This finding was somewhat be surprising because *in vitro,* bacterial killing by GEN was slightly faster than killing by CIN (Fig. 1). This discrepancy between *in vivo* and *in vitro* findings may be related to differences in the antibiotics’ distribution within the body. Ciprofloxacin is known to distribute extra- and intracellularly (especially within macrophages and polymorphonuclear cells), whereas the aminoglycosides are restricted to the extracellular space [25]–[27]. Although *Y. pestis* predominantly multiplies during infection as aggregates of extracellular bacteria, it can also replicate within host cells (such as macrophages). Indeed, intracellular *Y. pestis* has been observed during infection [10], [28]. Hence, our data may be due to GEN’s lower putative ability (relative to CIN) to kill intracellular *Y. pestis*.

Tissue conditions may also account for GEN’s relatively low efficacy in eradicating *Y. pestis* (especially from the draining lymph in surviving mice). Indeed, by 48 h after infection, it was previously reported that, the lymph node is mostly composed of necrotic tissue – with masses of *Y. pestis* mixed with cell debris and fibrin deposits and is characterized by low oxygen tension [10]. These anaerobic conditions reduce the activity of aminoglycosides but not that of fluoroquinolones [20], [29].

Regardless of the treatment and in contrast to the lymph node draining the injection site, the spleens from all surviving animals were sterile. This lack of detectable bacteria in the spleen in all three groups was probably not due to the absence of spleen colonization, since (i) 50% of the control animals in the present study developed terminal plague at the time of initiation of treatment (Table 1, group B) and (ii) it has been reported that spleen from rodents having succumbed to plague contained 10^8^ *Y. pestis* [10]. During the progression of bubonic plague, *Y. pestis* invades first the draining lymph node and then the spleen [9], [10]. Consequently, at the time of treatment, the bacterial load in spleen could have been lower than in the draining lymph node, which would have favored better sterilization activity in the spleen for all three regimens. Furthermore, less intense tissue alterations in the spleen as a result of a lower bacterial load may have improved the activity of GEN alone.

Lastly (and regardless of the treatment regimen), the lymph node may serve as a reservoir for *Y. pestis,* which can survive despite a 5-day course of i.v. treatment. Hence, the lymph node could also act as a source of subsequent *Y. pestis* release into the blood – an event that would result in fatal, secondary, septicemic plague. This hypothesis is notably supported by data from the GEN groups A and B, since (i) the lymph node from survivors could contain up to 7.6 log_10_ bacteria and (ii) two deaths were recorded after the end of the course of treatment. Here, the relapses observed in our GEN group corroborated literature results obtained in a mouse model of pneumonic plague, in which deaths occurred after the completion of a 5-day GEN regimen [7].

The survival rate for mice treated with CIN+GEN did not differ significantly from that for mice given CIN alone. Furthermore, the lymph node bacterial loads were similar in the CIN+GEN and CIN-only groups – indicating that the reduction in the bacterial load was due exclusively to CIN. However, two mice in the CIN group died during the treatment administration period starting 56 h after the challenge, whereas none of the animals in the CIN+GEN group died at this time point. Furthermore, kill-curves showed synergistic antibacterial activity at 24 h for low concentrations of GEN and CIN; this property could be beneficial during the blood dissemination of *Y. pestis*, especially when the antibiotics’ respective concentrations fall below the MIC. In blood, aminoglycoside activity should be optimal because of the aerobic environment and the extracellular location of *Y. pestis*. Lastly, aminoglycosides are known to reduce endotoxin release from large inocula of Gram-negative bacteria more effectively than fluoroquinolones do [30]. Thus, the use of CIN+GEN combination therapy in humans might help decrease post-septicemia mortality.

Our data revealed that the infected draining lymph node may serve as a reservoir for *Y. pestis* and thus enable the bacteria to survive and disseminate despite the initiation of antibiotic treatment. It also showed that regimens containing CIN alone or in combination are more effective than the recommended treatment with GEN alone to clear *Y. pestis* infection from the draining lymph node. The less sterilizing activity of GEN may explain the few relapses observed with the GEN monotherapy.
